# Cross‐cultural comparison of low back pain in the nursing workforce: A pilot study

**DOI:** 10.1111/ijn.13292

**Published:** 2024-08-12

**Authors:** Andrea Gilchrist, Denisa Macková, Andrea Pokorná

**Affiliations:** ^1^ Department of Health Sciences, Faculty of Medicine Masaryk University Brno Czech Republic; ^2^ Practice Development and Education, John Radcliffe Hospital Oxford University Hospitals NHS Foundation Trust Oxford UK

**Keywords:** general nurse, musculoskeletal low back pain, prevalence, prevention, risk factor

## Abstract

**Aim:**

This study aimed to analyse cross‐cultural differences in the prevalence of low back pain (LBP) and other back pain of general nurses in direct inpatient care in the Czech Republic (CZ) and Great Britain (GB).

**Methods:**

The survey was used using an extended standardized Nordic Musculoskeletal Questionnaire and self‐created additional questions. The data were analysed with Stata 15 using a significance level of 0.05.

**Results:**

The data analysis was based on 1043 questionnaires. We identified statistically significant differences in the LBP prevalence between the respondents (CZ 93% and GB 85%) over a period of 12 months. Nurses in both countries stated a higher prevalence of LBP compared to pain in the neck, shoulders or upper back. LBP increases in relation to age, length of work experience, body mass index (BMI) and university education (BSc). Age, length of work experience, BMI and education (nursing college and master's degree) were confirmed as significant risks contributing to the increased prevalence of other back pain as well. Respondents reported a reduction in work performance, leisure activities and the need to change profession in relation to LBP.

**Conclusion:**

The results of the study confirm that LBP is a frequent occupational health issue in the general nurses working in direct inpatient care in both countries.

## Summary statement

What is already known about this topic?

- The prevalence of LBP is more significant among nursing professionals and their aides than among women of the same age group in the general population, and studies have shown that LBP is a contributing factor to both recruitment and retention of nurses.
- Although injury prevention training for manual handling and the use of lifting equipment is a global competency and ergonomics management tools are considered important throughout the Western world, there is a lack of research and broad implementation of effective preventative measures that protect healthcare professionals musculoskeletal health in the Czech Republic and many other central European countries.
- Furthermore, lumbar spine injuries and illnesses are not acknowledged as occupational diseases by the Institute of Health Information and Statistics of the Czech Republic and many other countries across Europe. The prevalence of work‐related back pain and the incidence of lumbar spinal injuries among nursing staff have not been analysed at a nationwide level; thus, the number of health professionals who are seriously affected by musculoskeletal back pain in the Czech Republic is not known.

What this paper adds?

- This study on LBP prevalence in the general nurse population in the CZ and its further evaluation in the dimension of international comparison is the first to provide information on musculoskeletal health and LBP prevalence between countries with different implementation of measures for identifying the risk of musculoskeletal disease in healthcare professionals and for the prevention and protection of the health of the nursing workforce.
- This study underlines the value of the statutory listing of musculoskeletal disorders on the list of occupational diseases in order to provide an annual overview of the prevalence of spinal disorders and injuries and the origin of occupational accidents in specific occupations.
- Through the online distribution of a recognized robust tool—the standardized extended version of NMQ—we were able to reach out to a wide range of nursing workforce representatives to collaborate.

The implications of this paper:

- The results of the study provide unique fundamental quantitative data on the prevalence of LBP in the population of general nurses in direct inpatient care in the Czech Republic and Great Britain.
- The current research will be followed by a nationwide study that will involve nurses who have transitioned out of the healthcare industry prematurely or have pursued less strenuous job roles. This subsequent study aims to enhance our understanding of the effects of lower back pain and its impact on job retention.
- Information about the extent of nurses' musculoskeletal health is important for the global clinical community to understand its impact on nursing fluctuation and retention and to better address the issue of growing nursing shortages.

## 1 INTRODUCTION

Low back pain (LBP) is reported as one of the most common health problems, causing a rising number of sick absences and disability pensions in the general adult population across Europe (Laštovková et al., 2015). Langella et al. (2021), in their study, stated that the European Agency for Safety and Health at Work ranked musculoskeletal conditions as second in terms of causing disability‐adjusted life years, accounting for 16% overall in 2020. This is only surpassed by cancer, which accounted for 25% in the same year. Other researchers have reported that LBP is significantly higher in nurses than in women of the same age in the general population or in other healthcare professionals (Almaghrabi & Alsharif, 2021, p. 1567; D'Agostin & Negro, 2017, pp. 274–284; Fiter et al., 2018, pp. 1–8; Hartvigsen et al., 2005, pp. 13–17; Karahan & Bayraktar, 2013, pp. 73–78; Zhang et al., 2022). The literature review revealed that various factors, such as the physical requirements of the job and personal and psychosocial factors, can contribute to the occurrence of work‐related musculoskeletal disorders (Langella et al., 2021). Healthcare organizations worldwide are trying to tackle challenges caused by continuing nursing workforce shortages and to meet the growing demand for care for the ageing population (Kox et al., 2022). The post‐communist central European countries are particularly affected, as their numbers of nursing professionals have been reported below the European Union average (Gurková et al., 2020). An increasing amount of research has associated occupational LBP with high rates of absenteeism and early retirement from the profession (Brinkmann et al., 2022; Fiter et al., 2018, pp. 1–8; Simon et al., 2008, pp. 24–34) and has underlined negative socioeconomic impacts on individuals, families, communities and governments worldwide (Fiter et al., 2018, pp. 1–8; Souza et al., 2017; Yoshimoto et al., 2020; Zhang et al., 2022).

The analysis of LBP in association with loss of productivity in the nursing workforce varies in the literature worldwide. While Fiter et al. (2018, pp. 1–8) estimated that LBP is one of the most common reasons for nurses' absence from work, many studies have stated that nurses have ‘presenteeism’ (or ‘sickness presenteeism’), which is the situation when employees who complain of a deteriorating health condition that should prompt rest and absence from work reduce their productivity while remaining at work (d'Errico et al., 2013, pp. 276–283; Li et al., 2019; Skela‐Savič et al., 2017, pp. 544–551; Yoshimoto et al., 2020). Studies have shown that presenteeism associated with LBP negatively affects the quality of nursing professional life and safe patient care and that it is largely related to future productivity loss in terms of absenteeism (Li et al., 2019; Yoshimoto et al., 2020; Zhang et al., 2022).

## 2 BACKGROUND

Based on previous research, the development and persistence of work‐related musculoskeletal disorders are impacted by physical activity, working postures and working conditions within the workplace (Langella et al., 2021). Therefore, it is imperative to increase awareness, gather and provide access to reliable evidence in the respective field, execute comprehensive interventions for work health and safety, establish state policies to mitigate physical injuries at work, and underscore the significance of staff psychosocial well‐being and lifestyle factors (Brinkmann et al., 2022; Gilchrist & Pokorná, 2021a, pp. 1675–1683; Langella et al., 2021; Van Hoof et al., 2021).

In a previous study, Gilchrist and Pokorná (2021a, pp. 1675–1683) outlined the prevalence of LBP in a population of Czech nurses. They highlighted a lack of systematic data on the musculoskeletal health of the nursing workforce and insufficient compliance with health and safety regulations in Czech healthcare settings. This study aims to compare the previously published results of LBP in Czech nurses with research findings among a population of general nurses in direct inpatient care in Great Britain (GB).

We used an extended version of the Nordic Musculoskeletal Questionnaire 2 (NMQ‐E2) to assess the LBP prevalence in a selected population of general nurses practising in direct inpatient care in the Czech Republic (CZ) and in GB. In GB, there have been long‐implemented national preventive strategies and measures to prevent the occurrence of musculoskeletal pains in healthcare professionals, and therefore, the research is designed to compare the situation in both countries. The main practical aim of the study is to assess the cross‐cultural differences in LBP prevalence; such research may lead to increasing awareness and to the implementation of measures for identifying the risk of musculoskeletal disease in healthcare professionals and thus contribute to the prevention and protection of the health of general nurses. The presentation of the study findings was guided by the STROBE checklist for cross‐sectional study designs (see Table S1).

## 3 METHODS

### 3.1 Research methods

For this study, a quantitative research method was selected; specifically, a prospective cross‐sectional cohort survey study was conducted among general nurses in direct inpatient care in the CZ and GB. The data collected from both countries were compared to perform a comparative analysis. This study serves as a pilot for a future Czech national study surveying general nurses. The selection of participants was deliberate and purposeful, involving motivated general nurses with at least 1 year of experience in direct patient care who were willing to complete the questionnaire. The type of hospital ward was categorized by the author into three groups: general ward, intensive care unit (ICU) and urgent care/peri‐operative/post‐operative.

The primary requirement for participation in the study was a minimum of 1 year of professional clinical practice in direct inpatient care after completing the undergraduate professional education of a general nurse. Neither the maximum length of professional clinical experience nor the level of education attained were determined as limitation factors in the study.

### 3.2 Data collection tool

Based on a literature review, the NMQ‐E2 was selected and used with its author's approval to analyse the prevalence of LBP in nurses. The questionnaire originated from the standardized Nordic Musculoskeletal Questionnaire (NMQ) created by Kuorinka et al. (1987). The validity and reliability of the original pilot questionnaire were tested in 1987 on a sample of 25 general nurses who filled it out two times within 15 days. On average, 4.4% (between 0% and 4%) of the respondents gave opposing answers, and in one question, 25% of them provided inconsistent answers, which indicates a low level of validity and reliability (Gilchrist & Pokorná, 2021a, pp. 1675–1683).

Pugh et al. (2015) supplemented the multiply amended vision of the NMQ questionnaire in order to obtain information on the onset, prevalence and consequences of musculoskeletal LBP in healthcare professionals. The validity and reliability of NMQ‐E2 were tested by means of an online questionnaire on a set of 65 general nurses who also participated two times in the study. The questionnaire items had a high‐to‐medium strength of agreement and proportion of observed agreement; in ~75% of questions concerning prevalence and 99% of questions evaluating intensity and impact of pain, 10% and/or less disagreements were identified. In order to evaluate the expert agreement, the content validity index (CVI) was determined. For further tool validation, the individual content validity indexes (I‐CVI) were added up and divided by the number of items in order to obtain the summary average index (S‐CVI/Ave). The professionals had to agree with the content validity of items that equalled 1 (I‐CVI = 1.00). The summary validity index (S‐CVI‐Ave) had an acceptable value of ≥0.90. Five professionals confirmed the relevance and validity of the tool, with I‐CVI being 1.00 and S‐CVI‐Ave being 1.00 (Gilchrist & Pokorná, 2021a, pp. 1675–1683; Pugh et al., 2015, pp. 3550–3563).

The 2‐NMQ‐E2 contains a total of 155 questions concerning the occurrence of musculoskeletal disorders in nine anatomic areas: neck, shoulders, elbows, wrists, upper back, low back, one or both hips, one or both knees and one or both ankles/legs. For the purposes of the performed and presented study, we used only items related to pain localized in the low back (16 items in total). The standardized items analysing the LBP prevalence were supplemented by questions concerning the occurrence of musculoskeletal pain in other parts of the back and by social and demographic data collection and questions related to existing occupational health and safety principles and compliance therewith as reported by the general nurses (26 items in total). The severity of the reported musculoskeletal pain in the neck, shoulders, upper back and lower back was assessed using a pain scale consisting of 10 points. The scale measures the level of pain, with 0 indicating the absence of pain and 10 reflecting the most severe pain possible.

### 3.3 Data collection organization

Pilot studies were performed prior to starting a more extensive data collection at the beginning of 2019. The returnability of the pilot online questionnaires was 100% without any negative feedback or suggestions for changing the items in the questionnaire.

The data collection, described in detail in Table 1, was performed in both countries by means of an online survey in 2019 and 2020 (before the COVID‐19 pandemic). A follow‐up study detecting the changes after the COVID‐19 pandemic is ongoing.

**TABLE 1:** Data collection organization.

Country	Addressed	Completed	Rejected	Number of questionnaires analysed
CZ	1460	620	51	569
GB	1616	504	30	474

Abbreviations: CZ, Czech Republic; GB, Great Britain.

### 3.4 Ethical considerations

The author received ethical approval to conduct the survey in the CZ from the ethics committee (EC) of the Faculty of Medicine of Ostrava University in the CZ on 6 February 2019 (EC No. 02/2019). For the study in GB, ethical approval was obtained from the Clinical Trials and Research Governance, University of Oxford, Oxford University Hospitals NHS Foundation Trust. The study was approved as a ‘service evaluation of manual handling practice’ without being assigned an EC identification number.

### 3.5 Data processing procedure

The final set of respondents was analysed according to the previously described criteria for inclusion/exclusion from the study.

The accessible literature was studied to determine the potential correlations of LBP occurrence risk in general nurses. The existing correlations were grouped as (1) *nonmodifiable* (age, sex and education) and (2) *modifiable* (according to sports activity and safe behaviour during patient handling). The correlations between defined risk factors for LBP occurrence in the nursing workforce were measured according to bivariable and multivariable analyses. The LBP prevalence was recorded by the respondents for the preceding 12‐month period. The resulting data were subsequently compared to analyse the cross‐cultural differences in the LBP incidence in the nursing workforce in the CZ and GB. The statistical analysis was performed using Stata 15. The normality of variables was assessed using a visual evaluation of curves. A comparison of the groups for continuous variables was performed with a bilateral *t* test of an independent sample or by an analysis of variance (ANOVA) at the significance level of 0.05.

## 4 RESULTS

### 4.1 Characteristics of the study sample

The characteristic factors of both groups of general nurses are described in more detail in Table 2. In total, 569 nurses in the CZ and 474 nurses in GB participated in the study. Most respondents were women (CZ: 96%, *n* = 544; GB: 90%, *n* = 448). The average age, as well as the average number of years of service, differed for respondents in both countries. Even though the maximum attained age was higher in the group of nurses in GB (67 years), the average age in the group of nurses in the CZ (42 years) was higher by 4 years than in the group in GB (39 years); the calculated standard deviation was 9.61 in the CZ and 10.66 in GB. The average number of years of service of Czech nurses was 6 years higher than for the group of nurses in GB; the calculated standard deviation was 10.52 in the CZ and 11.64 in GB. When analysing the completed education levels, differences among respondents were found, whereas the type of employment relationship was similar in both countries.

**TABLE 2:** Demographic characteristics of respondents in both countries.

Demographic factors	General nurses in CZ (*n* = 569)	General nurses in GB (*n* = 474)
	*n* (%)	*n* (%)
Sex		
Female	544 (96%)	448 (90%)
Age		
Mean	42	39
Minimum	21	21
Maximum	65	67
Duration of professional clinical practice		
Mean	21	15
Minimum	1	1
Maximum	45	50
Professional competence		
Diploma (College of Nursing)	245 (43%)	133 (27%)
Diploma or associate degree^a^	122 (21%)	10 (2%)
Bachelor's degree in nursing	133 (23%)	249 (50%)
Master's degree in nursing	65 (11%)	102 (21%)
Doctoral degree in nursing	4 (1%)	3 (1%)
Type of ward (categorized)		
Standard ward	307 (54%)	373 (79%)
Intensive care unit	151 (27%)	56 (12%)
Urgent/preoperative and postoperative ward	111 (20%)	45 (9%)
Type of employment		
Full‐time employment	503 (88%)	384 (77%)
Part‐time employment	55 (10%)	92 (19%)
Combination of the above	11 (2%)	21 (4%)
Type of services (as per the number of working hours)		
12‐h shifts	413 (73%)	313 (63%)
8‐h shifts	156 (27%)	141 (28%)

Abbreviations: %, relative frequency; CZ, Czech Republic; GB, Great Britain; *n*, absolute frequency.

^a^ In the Czech education system, this level of education is reached at higher schools. For further comparison with other countries, it is called the diploma or associated degree in nursing.

### 4.2 Prevalence of LBP in comparison to musculoskeletal pain

**FIGURE 1:**
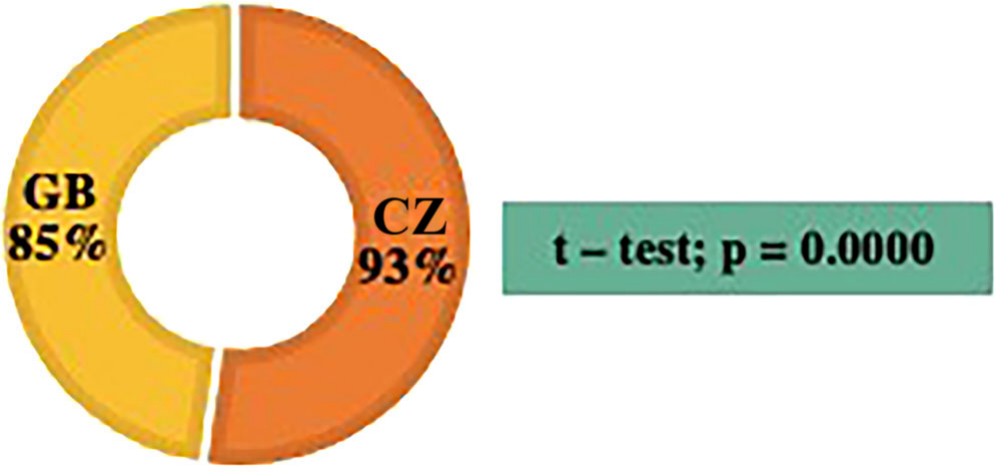
Cross‐cultural comparison of low back pain prevalence in the last 12 months. CZ, Czech Republic; GB, Great Britain.

From the analysis shown in Figure 1, it follows that most general nurses in both countries (in the CZ, 93%, i.e., *n* = 529 of the total number *n* = 569; in GB, 85%, i.e., *n* = 404 of the total number *n* = 474) stated an occurrence of LBP during the last 12 months (*t* test; *p* = 0.0000).

The intensity of the declared LBP was recorded by means of a 10‐point pain scale: 0 = *no pain*; 1–3 = *mild pain*; 4–6 = *moderate to severe pain*; 7–9 = *very severe pain*; and 10 = *described pain as bad as it could be*. An average score was calculated for musculoskeletal back pain intensity based on the data provided. The most commonly reported location for back pain was the lower back, with average scores of 1.800 in the CZ and 1.562 in GB. The calculated values for average scores of pain in the shoulders and upper back were similar, while the average score for pain in the neck area showed the greatest difference between the monitored countries (1.385 in the CZ and 0.966 in GB). It is worth noting that all average values indicate mild pain levels.

The average age for LBP occurrence in the group of nurses in the CZ (30 years) was almost no different from the average year of LBP occurrence calculated in the group of nurses in GB (29 years). LBP lasting for more than 30 days in the last 12 months was declared by 34% of nurses in the CZ and by 26% of nurses in GB. The group of nurses in GB most commonly stated LBP frequency at once in 7 days (GB 30% and CZ 26%). Everyday LBP occurrence was declared by 12% of nurses in the CZ and 15% of nurses in GB.

Most nurses in both countries stated that the back pain was not connected with any inborn defect, musculoskeletal disease or previous back injury (CZ 69% and GB 48%). When asked about a relationship between LBP occurrence and performance of the nursing profession, more than half of the respondents in both groups of nurses stated that, in their opinion, the LBP was partially caused by the performance of the nursing profession (CZ 59% and GB 58%); the option of a relation only with the profession was selected by 37% of the nurses in the CZ and 29% of the nurses in GB.

The analysis has demonstrably shown that LBP problems often resulted in a declared reduction of work activities (CZ 54% and GB 51%) and leisure time activities (CZ 73% and GB 52%) and, in some cases, also in a change of profession. The necessity to change the profession or work obligations performed as a consequence of LBP was stated by 34% of the group of nurses in GB and 19% of the nurses in the CZ.

Despite the fact that both sets of general nurses stated the necessity to seek medical assistance (CZ 55% and GB 39%) in relation to the back pain and an increase in the use of medication to manage the back pain (CZ 74% and GB 67%), only 6% of nurses in the CZ and 19% in GB declared a need to take sick leave during the last 12 months.

### 4.3 Sociodemographic factors associated with the prevalence of LBP

The analysis indicates that demographic and job characteristics (see Table 3) have no influence on the declared LBP prevalence when comparing the sets in the respective country. In the category of taking sick leave, there are differences when comparing the findings among the respondents in both countries, and a statistically significant difference was proved in the set of general nurses in GB (*p* = 0.0498); in the group of nurses in the CZ, the difference was not proved (*p* = 0.4200).

**TABLE 3:** Demographic factors in relation to LBP prevalence during 12 months—according to the country of performing the profession.

Demographic factors	Country	Cramér's *v*	*p*
Age	CZ	−0.0547	0.2092
	GB	−0.0401	0.4217
Body mass index (BMI)	CZ	0.0259	0.5520
	GB	0.0712	0.1531
Education	CZ	0.0016	0.9712
	GB	0.0153	0.7593
Sport	CZ	0.0010	0.9817
	GB	−0.0183	0.7145
Duration of work experience	CZ	−0.0303	0.4867
	GB	−0.0045	0.9284
Employment relationship	CZ	−0.0380	0.3833
	GB	0.0014	0.9770
Type of services	CZ	0.0062	0.8885
	GB	−0.0056	0.9149
Taking sick leave	CZ	0.0357	0.4200
	GB	0.0990	0.0498

Abbreviations: CZ, Czech Republic; GB, Great Britain; LBP, low back pain.

For completeness, we performed a more detailed analysis and tested the statistical significance for each sociodemographic category separately (see Table 4). When studying the declared back pain in detail in relation to sociodemographic characteristics, we found statistically significant differences in the age groups (21–29 years, *p* = 0.0316; 30–49 years, *p* = 0.0011), in the duration of practice (0–9 years, *p* = 0.0070), in body mass index (BMI) values (20–24.9, *p* = 0.0001; 25–29.9, *p* = 0.0382) and in university bachelor's degree education (*p* = 0.0340); in general, respondents in the CZ declared LBP prevalence more often than those in GB.

**TABLE 4:** Sociodemographic variables depending on LBP prevalence over 12 months—comparison between countries.

Sociodemographic variables	Country		*t* test
	CZ Percentage with pain	GB Percentage with pain	*p*
Age (years)			
21–29	90.9%	78.5%	0.0316
30–49	94.4%	86.7%	0.0011
50–59	91.1%	91.0%	0.9882
60+	78.6%	77.8%	0.9661
Work experience (years)			
0–9	91.2%	78.3%	0.0070
10–24	93.1%	88.5%	0.1304
25+	93.2%	94.3%	0.6974
BMI			
−19.9	87.5%	80.0%	0.4500
20–24.9	93.4%	79.7%	0.0001
25–29.9	91.9%	84.7%	0.0382
30–34.9	95.8%	90.3%	0.1785
35+	93.8%	96.0%	0.7121
Education			
Diploma (College of Nursing)	93.5%	90.5%	0.3021
Diploma or associate degree	94.1%	100.0%	0.5111
Bachelor's programme	91.5%	83.2%	0.0340
Master's programme	89.7%	83.9%	0.3210
Doctoral programme	100.0%	33.3%	0.0624

Abbreviations: BMI, body mass index; CZ, Czech Republic; GB, Great Britain; LBP, low back pain.

When analysing other types of back pain declared by the respondents in relation to sociodemographic characteristics, we found differences in age groups (30–49 years, *p* = 0.0000; 50–59 years, *p* = 0.0008; 60+ years, *p* = 0.0183), years of service (10–24 years, *p* = 0.0002; 25+ years, *p* = 0.0000), BMI values (20–24.9, *p* = 0.0022; 25–29.9, *p* = 0.0137; 30–34.9, *p* = 0.0000) and completed education level (diploma [College of Nursing], *p* = 0.0000; master's degree, *p* = 0.0470); the average prevalence shows that respondents in the CZ declared prevalence of other musculoskeletal back pain more often—for more details, see Table 5.

**TABLE 5:** Sociodemographic variables in relation to prevalence of other musculoskeletal back pain over 12 months—comparison between the countries.

Sociodemographic variables	Country		*t* test
	CZ	GB	
	Average prevalence of other back pain		*p*
Age (years)			
21–29	2.18	2.26	0.7331
30–49	2.56	2.09	0.0000
50–59	2.69	2.09	0.0008
60+	2.93	1.56	0.0183
Work experience (years)			
0–9	2.22	2.19	0.8814
10–24	2.59	2.08	0.0002
25+	2.65	2.06	0.0000
BMI			
−19.9	2.16	1.72	0.1843
20–24.9	2.61	2.19	0.0022
25–29.9	2.46	2.09	0.0137
30–34.9	2.94	2.10	0.0000
35+	2.06	2.24	0.6120
Education			
Diploma (College of Nursing)	2.68	1.94	0.0000
Diploma or associate degree	2.70	2.71	0.9809
Bachelor's programme	2.13	2.18	0.7132
Master's programme	2.62	2.19	0.0470
Doctoral programme	2.50	2.00	0.7015

Abbreviations: BMI, body mass index; CZ, Czech Republic; GB, Great Britain.

## 5 DISCUSSION

In our literary research, we verified that the issue of LBP prevalence in the nursing workforce, in spite of the options offered by modern technologies, is a lasting and very serious problem affecting 61%–79% of the responding nurses (Gilchrist & Pokorná, 2020, pp. 193–199). Because the healthcare industry requires employees to perform manual work, general nursing remains the most dangerous occupation, causing low back disability and injury (Brinkmann et al., 2022; Fiter et al., 2018, pp. 1–8; Gilchrist & Pokorná, 2020, pp. 193–199; Simon et al., 2008, pp. 24–34).

In the CZ, the issue of health protection and the impact of overworking of healthcare workforce in direct patient care are still insufficiently discussed. Despite the fact that general nurses are usually the most important workforce in health care, there is no systematic data collection concerning their state of health in the CZ (Gilchrist & Pokorná, 2021a, pp. 1675–1683).

### 5.1 Characteristics of the study sample

We analysed statements from 569 nurses in the CZ and 474 nurses in GB who participated in the research upon fulfilment of the inclusion criteria. Similar to the studies conducted by Skela‐Savič et al. (2017, pp. 544–551), June and Cho (2011, pp. 479–487) and Ribeiro et al. (2017, pp. 72–77), most participants in our study were women (CZ 96% and GB 90%). This may be consistent with the general assumption of the feminisation of the nursing profession. The observed value of average age (CZ 42 years and GB 39 years) corresponds with the results of other international studies: 40.8 years (Skela‐Savič et al., 2017) and 39.5 years (Ribeiro et al., 2017, pp. 72–77). The average period of nursing work experience of our respondents (CZ 21 years and GB 15 years) was longer than in other studies: 14.36 years (Skela‐Savič et al., 2017) and 17 years (D'Agostin & Negro, 2017, pp. 274–284).

During the study, both groups of respondent nurses were working mostly full‐time/main employment (88% in CZ and 77% in GB) and 12‐h shifts (73% in CZ and 63% in GB). When analysing the level of education achieved by the respondents, we found that university education was predominant in the group of nurses in GB (72%), in contrast to nurses in the CZ (36%). The differences in the education level reflect that before the CZ joined the European Union in 2004, the education system for general nurses in the CZ was different from the system used abroad. Since 1999, when the Bologna Declaration was adopted by 47 European and non‐European countries that agreed to increase the accessibility, attractiveness and quality of university education, the professional preparation of nurses has gradually been transferred to university institutions (Collins & Hewer, 2014; Lahtinen et al., 2014; Satu et al., 2013, pp. 625–632). Before 2004, general nurses in the CZ could only acquire professional competence at nursing colleges (Tóthová & Sedláková, 2008, pp. 33–38). In 2013, GB ruled that all new nurses would only become professionally competent after they completed an accredited study programme at universities (Shields & Watson, 2007).

### 5.2 Prevalence of LBP in comparison to musculoskeletal pain in the other back regions

An analysis of the standardized questionnaire has shown that a majority of general nurses in both countries (CZ 93% and GB 85%) described a prevalence of LBP in a 12‐month period. The CZ sample of nurses described LBP more frequently than the GB sample; there are statistically significant differences in the prevalence of LBP among general nurses practising in direct inpatient care in the CZ and GB (*p* = 0.0000).

By calculating the average musculoskeletal back pain intensity score on a scale of 1 to 10, it has been found that both groups of nurses described a demonstrably higher incidence of musculoskeletal pain in the low back than in the neck, shoulders or upper back. This is consistent with previous studies on the prevalence of LBP conducted between 2006 and 2017, in which LBP also proved to be the most common back pain issue. For example, a high prevalence of LBP was found in the nursing workforce in continental Europe: 85.9% in Slovenia (*n* = 1744) (Skela‐Savič et al., 2017); 76% in the Netherlands (*n* = 3169) (Bos et al., 2007, pp. 198–206), 75% in Greece (*n* = 351), 46% in Ireland (*n* = 246) (Cunningham et al., 2006, pp. 447–454) and 60.9% in Portugal (*n* = 1396) (Serranheira et al., 2015, pp. 401–409). Studies of LBP prevalence in non‐European regions have been conducted in Taiwan (72.0%; *n* = 567) (Shieh et al., 2016, pp. 525–529), Japan (71%; *n* = 844) (Smith et al., 2006, pp. 195–200) and Saudi Arabia (82.9%; *n* = 234) (Almaghrabi & Alsharif, 2021, p. 1567).

### 5.3 Sociodemographic factors associated with the prevalence of LBP

Samaei et al. (2017, pp. 551–561) and Nelson et al. (2003, pp. 32–43) confirmed the association of LBP with age, BMI, sex, number of hours worked per week, work in shifts and duration of employment. The results of our study demonstrated relations between declared LBP prevalence and age (21–29 years, *p* = 0.0316; 30–49 years, *p* = 0.0011), work experience (0–9 years, *p* = 0.0070), BMI values (20–24.9, *p* = 0.0001; 25–29.9, *p* = 0.0382) and university bachelor's degree (*p* = 0.0340) when comparing the two countries.

When analysing other back pain declared by the studied population of nurses in relation to sociodemographic characteristics, we found differences between the countries in age groups (30–49 years, *p* = 0.0000; 50–59 years, *p* = 0.0008; 60+ years, *p* = 0.0183), years of service (10–24 years, *p* = 0.0002; 25+ years, *p* = 0.0000), BMI values (20–24.9, *p* = 0.0022; 25–29.9, *p* = 0.0137; 30–34.9, *p* = 0.0000) and completed education (secondary school, *p* = 0.0000; university master's degree, *p* = 0.0470).

Serranheira et al. (2015, pp. 401–409) studied the impact of nursing interventions and found that invasive nursing acts, assistance with feeding and hygiene, and patient manipulation represent a very significant work risk that largely contributes to LBP in the nursing profession. In another study, Skela‐Savič et al. (2017) stated that job dissatisfaction as a result of insufficient job evaluation, regular sports activities and level of education was negatively related to LBP in nurses. Contemporary literature clearly indicates that frequent overtime work and short recovery times between individual shifts lead to overload and fatigue, resulting in slowed critical judgement and reduced motivation to follow health and safety regulations at the clinical workplace (Abdul Rahman et al., 2017, pp. 13–18). Shieh et al. (2016, pp. 525–529) calculated that the risk of LBP increases by 35% per every hour worked beyond the 9‐h shift. As the length of shifts is one of the main factors linked with LBP prevalence in many foreign studies, we need to consider a recommendation to shorten the 12‐h working shifts and, at the same time, replace manual lifting of patients with appropriate mechanical aids.

### 5.4 Evaluation of level of LBP prevalence impact

The results of our research have shown that the impacts of LBP stated by the respondents include a reduction in work activities (CZ 54% and GB 51%), leisure activities (CZ 73% and GB 52%) and, in some cases, also a need to change profession. Despite the finding that both groups of nurses mostly described a need to seek medical assistance (CZ 55% and GB 39%) and an increase in using medication to manage the back pain (CZ 74% and GB 67%), only 6% of nurses in the CZ and 19% in GB stated that they had needed to take sick leave during the last 12 months. A statistically significant connection between LBP and taking sick leave was only confirmed in the set of nurses in GB (*p* = 0.0498); in the group of nurses in the CZ, no difference was confirmed (*p* = 0.4200).

The results of the analysis of the effect of LBP on taking sick leave in the nursing profession vary in the international literature. Fiter et al. (2018, pp. 1–8) assumed that LBP is one of the most common reasons for nurses' absence from work; plenty of foreign sources state that in the general nursing profession, there is the so‐called sickness presenteeism, a notion used to describe situation when employees who complain of a deteriorating health condition that should prompt rest and absence from work reduce their productivity while remaining at work (d'Errico et al., 2013).

In general, the impact of LBP on the incapacity to work is a frequently discussed topic in Czech and foreign literature. Nevertheless, musculoskeletal diseases do not rank first in the annual national analysis of the most common causes of incapacity to work in the Czech population. In contrast to the most reported respiratory diseases, lasting 2 weeks on average (Bosák & Másilková, 2018), temporary work incapacity due to diseases of the musculoskeletal system lasts on average for more than two calendar months (66 days) per case. Musculoskeletal disease accounted for one third of the total number of days lost due to sickness, the highest share in a long time, and musculoskeletal diseases clearly prevail (Occupational Safety Research Institute [CZ], 2019). The seriousness of the issue of high dorsalgia prevalence is also reflected in the analysis of reported cases of disability, which very often logically follow a long‐term incapacity to work. When assessing a patient's state of health for the purposes of determining disability, musculoskeletal diseases are the most frequent cause of recognition of disability of the first and second degree in the Czech population. The analysis shows that, in particular, dorsalgia, polyarthralgia, coxarthrosis and gonarthrosis are listed as the most common primary diagnoses (Bosák & Másilková, 2018). From a retrospective analysis of healthcare registries in the epidemiology of dorsalgia and other intervertebral disc diseases in the general population in the CZ between 2010 and 2018, we found that the total number of patients with dorsalgia and intervertebral disc disease showed an increasing tendency. Nevertheless, given the data available, it is not possible to obtain an overview of musculoskeletal disorders in relation to occupation (Gilchrist & Pokorná, 2021b).

### 5.5 Strengths and limitations

This study on LBP prevalence in the general nurse population in the CZ and its further evaluation in the dimension of international comparison is the first to provide information on musculoskeletal health and LBP prevalence. Through the online distribution of a recognized robust tool—the standardized extended version of NMQ—we were able to reach out to a wide range of nursing workforce representatives to collaborate. The survey forms were only filled out by motivated respondents with an interest in the topic.

Questionnaire surveys tend to be limiting as they generally yield rather subjective views of respondents, but by maintaining anonymity, providing answers to sensitive questions relating to musculoskeletal health and the impact of LBP on general nurses' everyday lives was guaranteed.

A limiting factor of the study was the restriction of the research to nursing staff currently working in the CZ and GB, which meant that nurses who had left or changed direct care professions were not included in the study. As a result, the seriousness of LBP prevalence in general nurses in direct inpatient care in the CZ and GB may have been underestimated.

We also recognize the importance of conducting a more thorough analysis of various factors that contribute to the occurrence or continuation of lower back pain (LBP) from a biopsychosocial perspective. The present research will be followed by a nationwide investigation aimed at exploring additional influential aspects that are equally relevant to the nursing profession.

### 5.6 Relevance to clinical practice

The results of the present study provide unique fundamental quantitative data on the prevalence of LBP in the population of general nurses in direct inpatient care in the CZ and GB, and the present study will be followed by a national study that will include nurses who have left the healthcare profession early or changed their job to a less demanding one, which will enable a better understanding of the impact of LBP.

Healthcare organizations or researchers studying the LBP issue can use the validated Czech version of the NMQ‐E2 standardized questionnaire, which is available as a tool for assessing LBP through this study.

Other studies should be conducted using a prospective observational study methodology to identify the high‐risk patient handling behaviour of general nurses and the availability and correct use of positioning and handling equipment in clinical practice.

Finally, we would like to highlight the importance of the statutory listing of musculoskeletal disorders on the list of occupational diseases in order to provide an annual overview of the prevalence of spinal disorders and injuries and the origin of occupational accidents in specific occupations.

## 6 CONCLUSION

The results of the study suggest that general nurses in the CZ group of responding nurses, as compared to those in GB, are more likely to report having a high prevalence of LBP.

Consistent with previous studies, we found that the prevalence of musculoskeletal LBP was most frequently reported, with pain in other parts of the back being less frequently reported. The influence of sociodemographic characteristics and employment‐related factors in relation to the prevalence of LBP is consistent with the results of other international studies. We confirmed that LBP increases in relation to the respondent's age, length of work experience, BMI and university education (BSc). When analysing the other back pain reported by respondents, age, length of experience, BMI and education (secondary school and master's degree) were confirmed as highly significant risks contributing to the increased prevalence of other back pain.

The impact of LBP on psychosocial areas of nurses' lives was found to be consistent with previous research and assessed as significant. Respondents in our study reported a reduction in work performance and in the volume of activities performed, including leisure activities; in some cases, they reported a need to change profession in relation to the occurrence of LBP.

The analysis of the relationship between LBP and increased sick leave does not correspond with the results of international studies. The present research was limited to currently employed nursing staff, not including general nurses who left or changed profession due to back pain or other health issues. As a result, the research team is presently working to conduct follow‐up research to provide a more in‐depth overview of musculoskeletal health and the impact of workload in the health sector as factors influencing health worker turnover and retention.

## AUTHORSHIP STATEMENT

**Andrea Gilchrist:** Conceptualization; methodology; data collection and extraction; interpretation of data; data analysis; project administration; writing—original draft; final approval of the version to be submitted. **Denisa Macková**: Conceptualization; methodology; data collection and extraction; interpretation of data; writing—original draft; final approval of the version to be submitted. **Andrea Pokorná:** Conceptualization; methodology; data collection and extraction; interpretation of data; writing—review and editing; supervision; final approval of the version to be submitted.

## CONFLICT OF INTEREST STATEMENT

The authors declare no conflict of interest.

## ETHICAL CONSIDERATIONS

The author obtained consenting opinions to perform the survey from the ethics committee (EC) of the Faculty of Medicine of Ostrava University in the Czech Republic on 6 February 2019 (EC No. 02/2019) and from the Clinical Trials and Research Governance, University of Oxford, Oxford University Hospitals NHS Foundation Trust. The study was approved as a ‘service evaluation of manual handling practice’ without awarding an EC identification number.

## Supporting information


**Table S1.** STROBE Statement—checklist of items that should be included in reports of observational studies.

## Data Availability

The data that support the findings of this study are available from the corresponding author upon reasonable request.
